# Oral 5-Aminosalicylate, Mesalamine Suppository, and Mesalamine Enema as Initial Therapy for Ulcerative Proctitis in Clinical Practice with Quality of Care Implications

**DOI:** 10.1155/2016/6928710

**Published:** 2016-04-12

**Authors:** James M. Richter, Nabeela K. Arshi, Gerry Oster

**Affiliations:** ^1^Massachusetts General Hospital and Harvard Medical School, Boston, MA 02114, USA; ^2^Policy Analysis Inc., Brookline, MA 02445, USA

## Abstract

*Background.* Ulcerative proctitis (UP) is typically treated initially with oral 5-aminosalicylate (“5-ASA”), mesalamine suppository, or mesalamine enema (“UP Rx”). Little is known about their effectiveness in practice.* Methods.* Using a US health insurance database, we identified new-onset UP patients between January 1, 2005, and December 31, 2007, based on the following: (1) initiation of UP Rx; (2) endoscopy in prior 30 days resulting in diagnosis of UP; and (3) no prior encounters for ulcerative colitis or Crohn's disease. We examined the incidence of therapy escalation and total costs in relation to initial UP Rx.* Results.* We identified 548 patients: 327 received mesalamine suppository, 138 received oral 5-ASA, and 83 received mesalamine enema, as initial UP Rx. One-third receiving oral 5-ASA experienced therapy escalation over 12 months, 21% for both mesalamine suppository and enema. Mean cumulative total cost of UP Rx over 12 months was $1552, $996, and $986 for patients beginning therapy with oral 5-ASA, mesalamine enema, and mesalamine suppository, respectively. Contrary to expert recommendations the treatments were often not continued prophylactically.* Conclusions.* Treatment escalation was common, and total costs of therapy were higher, in patients who initiated treatment with oral 5-ASA. Further study is necessary to assess the significance of these observations.

## 1. Introduction

Ulcerative colitis is a common chronic idiopathic inflammatory disease of the colon, which causes diarrhea, bleeding, and pain; its severity can range from mild to life-threatening. Ulcerative colitis typically extends proximally in a circumferential and uninterrupted manner that involves either all or only parts of the colon and sometimes is referred to with different terms, such as left-sided or pan colitis, depending upon the degree of colonic involvement. In approximately 30% of patients, the disease presents as ulcerative proctitis [1, 2] with bowel inflammation limited to the rectum. Approximately 50% of patients with ulcerative proctitis have a relapsing-remitting course that is characterized by flares of varying severity and periods of remission during which patients are relatively asymptomatic. During flares, patients typically experience rectal bleeding, discomfort and urgency, pain, passage of mucous, tenesmus, and diarrhea.

In general, treatment of ulcerative proctitis is determined by the severity of symptoms and the extent of colonic involvement [3–6]. Therapy is customarily tailored to the needs of individual patients and is designed to reduce inflammation, moderate symptoms, and maintain remission once achieved. Treatment usually begins with oral mesalamine or rectally administered (i.e., topical) mesalamine or glucocorticosteroids.

The efficacy of these drugs has been demonstrated in randomized controlled trials. Most such studies, however, are placebo-controlled and do not involve active comparators; relatively little is therefore known about the comparative effectiveness of drugs that form the cornerstone of the treatment of ulcerative proctitis. Those few head-to-head clinical trials that have been conducted point to possible differences in effectiveness that may be important in clinical practice. For example, a small randomized trial of oral versus rectal mesalamine by Gionchetti and colleagues in 58 patients, aged >18 years, with active, histologically confirmed ulcerative proctitis, demonstrated the superiority of rectal drug administration, in terms of improvement in mean Disease Activity Index (DAI) score (*p* < 0.001), as well as the rate of histologic remission (*p* < 0.01) [7]. A similar trial by Kam and colleagues reported equivalent efficacy for oral versus rectal administration of mesalamine in terms of the primary efficacy outcome (DAI score), but rectal administration reportedly was found to be superior on a number of secondary efficacy endpoints, such as both physician-rated and patient-rated global improvement. The percentage of patients experiencing adverse events also was higher in the oral mesalamine group [8]. Another trial by Biddle and colleagues found that the efficacy of oral and rectal mesalamine did not differ but reported that combined treatment was superior to either drug administered alone [9]. Because the number of head-to-head comparisons of drug therapies for the treatment of ulcerative proctitis is small, we undertook a retrospective study to examine the comparative effectiveness of oral 5-aminosalicylate (“oral 5-ASA”), mesalamine suppository, and mesalamine enema as initial therapy in patients newly presenting in clinical practice with ulcerative proctitis.

## 2. Materials and Methods

### 2.1. Data Source

This study used data from a large, United States based private health insurance claims database, Thomson Reuters MarketScan Commercial Claims and Encounters and Medicare Supplemental and Coordination of Benefits Database (“MarketScan database”), spanning the period of January 1, 2004, through December 31, 2008. The database consists of information from employer-sponsored insurance plans throughout the US providing health benefits to over 15 million persons annually, including employees, their spouses, and their dependents.

Data available for each facility and professional-service claim include date and place of service, diagnoses, procedures performed/services rendered, and quantity of services (professional-service claims). Data available for each retail pharmacy claim include the drug dispensed, dispensing date, quantity dispensed, and number of therapy days supplied. Selected demographic and insurance eligibility information is also available for persons in the database, including age, sex, geographic location, coverage type, and the beginning and end (if relevant) of health insurance coverage. All claims include paid (i.e., reimbursed) amounts, including patient deductibles, copays, and/or coinsurance amounts. Patient-level data can be arrayed chronologically to provide a detailed longitudinal profile of all medical and pharmacy services used by each plan member.

### 2.2. Sample Selection

We identified all persons in the study database aged ≥18 years who underwent colonoscopy or sigmoidoscopy leading to a diagnosis of ulcerative proctitis (ICD-9-CM 556.2) or ulcerative proctosigmoiditis (556.3) between January 1, 2005, and December 31, 2007. Among these persons, we identified those who also had evidence of receipt of an oral 5-ASA (balsalazide, oral mesalamine (e.g., Asacol®, Pentasa®, and Rowasa®), and sulfasalazine), mesalamine suppository (Canasa®), or mesalamine enema (e.g., Rowasa) (“UP-related medications”) within 30 days of the procedure. Date of initial receipt of UP-related medication was designated the “index date.”

We excluded patients with (1) less than 12 months of complete encounter data prior to and following their index date (i.e., 24 months in total); (2) evidence of receipt of 5-ASA, metronidazole, antimetabolite, glucocorticoid, or TNF inhibitor within 365 days prior to their index date; (3) any medical encounters with a primary diagnosis code of ulcerative colitis (ICD-9-CM 556.X, excluding 556.2 and 556.3) and/or Crohn's disease (ICD-9-CM 555.X or 556.X) in the period beginning 365 days prior to index date and ending 31 days prior to index date; or (4) evidence of receipt of more than one UP-related medication on their index date. We then stratified patients into three mutually exclusive treatment groups, based on the therapy received on the index date, as follows: (1) oral 5-ASA; (2) mesalamine suppository; or (3) mesalamine enema.

### 2.3. Measures and Analyses

We examined the baseline characteristics of patients in each treatment group, including age, gender, geographic location, principal payer, and therapy days dispensed on the index date. Comorbidities were assessed based on a scan of all ICD-9-CM diagnosis codes in the one-year period preceding the index date.

Measures that we examined over the one-year period following each patient's index date included the following: (1) receipt of any other medication for the treatment of inflammatory bowel disease (IBD) (e.g., corticosteroids, immunomodulators, and biologic response modifiers) (“treatment escalation”); (2) hospitalization and emergency department encounters for the treatment of IBD; and (3) total costs of pharmacotherapy for IBD. Each of these measures was examined on a cumulative basis, beginning with the index date. All analyses were descriptive in nature, and significance testing was not performed, as there were no a priori hypotheses. All costs are expressed in United States dollars.

## 3. Ethical Considerations

All Protected Health Information (as defined by the Health Insurance Portability and Accountability Act of 1996 and federal guidance on Public Welfare and the Protection of Human Subjects) was either removed from the database or encrypted, rendering it a “limited dataset” containing only “deidentified” information, as defined by relevant legislation and regulation [10, 11]. Institutional Review Board (IRB) review was not needed for this study, per the Code of Federal Regulations, since “…subjects cannot be identified, directly or through identifiers linked to the subjects…” [11].

## 4. Results

### 4.1. Patient Characteristics

We identified a total of 548 patients who met all study entry criteria; 145 patients were excluded due to evidence of receipt of >1 UP-related medication on index date (Table 1). Patient mean (±SD) age was 47.3 (±15.6) years, and the number of men and women was approximately equal. More than 40 percent of study subjects were from the South (US), and over one-half (54.4%) had health insurance coverage characterized by a preferred provider organization. Hypertension, diabetes, and heart disease were frequently noted comorbidities, based on a scan of ICD-9-CM diagnosis codes in the year prior to index date (Table 2). The most frequently prescribed initial therapy was mesalamine suppository (59.7% of study subjects), followed by oral 5-ASA (25.2%) and mesalamine enema (15.1%).

### 4.2. Treatment Escalation

**Figure 1:**
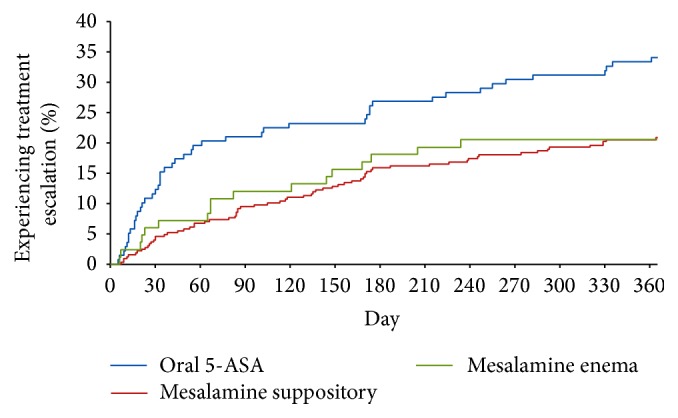
Cumulative percentage of study subjects experiencing treatment escalation, by duration of follow-up and treatment group. Oral 5-ASA: oral 5-aminosalicylate.

The percentage of patients who experienced treatment escalation (i.e., began therapy with another medication for IBD, such as a corticosteroid, immunomodulator, or biologic response modifier) was consistently higher among those who received oral 5-ASA on their index date in comparison with those who received mesalamine suppository or mesalamine enema as their initial therapy (Figure 1). At one year, 34.1% of patients who initiated treatment with oral 5-ASA had evidence of receipt of another agent, versus 20.8% and 20.5% of patients who received mesalamine suppository or mesalamine enema, respectively, on their index date (Table 3).

### 4.3. Hospitalizations and Emergency Department Visits

Very few patients were hospitalized for the treatment of IBD over the one-year period of follow-up (Table 4); rates of emergency department encounters also were low (Table 5). No patients underwent surgery for IBD during this 12-month period.

### 4.4. Cost of Pharmacotherapy

Mean cumulative cost per patient of pharmacotherapy for the treatment of IBD increased over time in all three treatment groups. At one year, mean cumulative cost of pharmacotherapy for IBD in patients who began treatment with oral 5-ASA averaged $1552; for patients initiating treatment with mesalamine enema, mean cumulative cost was $995; and for those who received mesalamine suppository on their index date, mean cumulative cost per patient was $986 (Table 6). There were no meaningful differences in total costs among the three groups.

### 4.5. Quality of Care Implications

Among the patients with ulcerative proctitis beginning therapy with a mesalamine suppository, approximately 70% discontinued such treatment within one month; about 20% of these patients, however, had evidence of continued receipt of mesalamine suppositories throughout the year. While adjunctive use of oral medications, mainly, oral 5-ASAs, was low initially (4%), it increased steadily over the year.

## 5. Discussion

Treatment of ulcerative proctitis usually begins with oral mesalamine or rectally administered (i.e., topical) mesalamine or glucocorticosteroids. Although the efficacy of these drugs has been demonstrated in randomized controlled trials, they rarely have been compared on a head-to-head basis, and evidence of their comparative effectiveness is therefore limited. To shed light on this issue, we undertook a retrospective study of the effectiveness of oral 5-ASA, mesalamine suppository, and mesalamine enema as initial therapy in patients newly presenting in clinical practice with ulcerative proctitis.

Our findings confirm the relatively favorable natural history of distal colitis in a large community-based cohort of patients with newly presenting disease who were followed up for one year. There were few hospitalizations, and no one required surgery or was treated with a biological agent. Our results also confirm the observation that topical or rectal mesalamine may be superior to oral mesalamine. Although we measured treatment escalation rather than response to treatment, which is the more commonly studied outcome, it is a complementary endpoint [12]. Treatment escalation in clinical practice represents the active judgment of the treating physician that prior treatment was unsatisfactory. We could not determine if the treatment escalation was due to disease extension. In addition, it is a comparison of treatment options, rather than a comparison of a treatment to placebo, and addresses the relevant question of comparative effectiveness of treatments that practicing clinicians confront.

Interestingly, despite our results and previous studies demonstrating the superiority of topical mesalamine for patients with limited disease, the frequency of prescribing oral mesalamine increased sixfold between 1992 and 2009, while the frequency of prescribing topical preparations was almost unchanged [13]. Nevertheless, the majority of patients with ulcerative proctitis in our study were treated with topical drugs. To the best of our knowledge, our study is the first to utilize health insurance claims data, and our results suggest that topical therapy may be less costly and more effective than the other treatment options studied.

The American College of Gastroenterology and the Toronto Consensus recommend that 5-ASA preparations be used as initial treatment for patients with mild to moderate disease and for the prevention of relapse; in patients with disease confined to the rectosigmoid area, topical preparations may be useful [3, 14]. The failure to continue treatment for prevention represents a deviation from recommendations, might lead to later escalation, and deserves further study. The relative convenience, acceptability of, and compliance with oral versus rectal dosing have not been systematically studied. We did not directly measure compliance in our study, but claims data are based on dispensed prescriptions rather than physician orders, and the former may serve as a proxy for treatment compliance. One hundred forty-five patients were treated with combinations that prevented us from studying those subgroups; findings from work by Safdi and colleagues suggest that treatment with the combination of oral and rectal mesalamine therapy may provide faster and more complete symptom relief than either therapy alone [15].

We note a number of limitations of our study. As with all studies based on administrative databases, errors of commission and omission are always a concern, especially regarding the ascertainment of medical conditions using ICD-9-CM diagnosis codes. The sensitivity and specificity of such codes in correctly identifying patients with IBD were recently reported to be 84% and 99%, respectively [16]. We also note that since data were left-censored 12 months prior to index date, our study population probably consisted of patients with true incident cases as well as those experiencing recurrence of their disease. This could also potentially serve as an explanation for some of the colorectal and digestive comorbidities recorded at baseline and could introduce selection bias—specifically, lead-time bias—since comparisons between patients and treatment groups would no longer be based on the same point in the course of disease. Additionally, our analysis may not be representative of the entire population of patients with ulcerative proctitis, due to the underrepresentation of older Medicare patients in our database and the exclusion of children. Next, although we examined some baseline characteristics, there may be inherent, unnoted differences (e.g., lifestyle and diet) between patients in the three treatment groups or between the types of disease (e.g., proctitis and proctosigmoiditis) that patients experienced in each treatment group, which may affect outcomes. The impact of the treatments on recurrence rates, disease progression, or the need for prophylactic treatment is also unknown and was not determined in our analyses. Similarly, the data do not provide insight into patient treatment adherence or reasons for treatment escalation, which may or may not be related to the initial therapy itself. Finally, the lack of significance testing means that we cannot make definitive statements about the significance of differences between treatment groups.

In summary, our study suggests that treatment escalation may occur more frequently among patients with new-onset ulcerative proctitis who initiate treatment with oral 5-ASA rather than mesalamine suppository or mesalamine enema. Total costs of pharmacotherapy also may be higher in patients receiving oral 5-ASA as initial therapy. Further study is necessary to assess the significance of these observations.

## Figures and Tables

**Table 1:** Selection of study subjects with newly diagnosed ulcerative proctitis.

Criterion	Total patients
Patients aged ≥18 years who underwent colonoscopy/sigmoidoscopy between January 1, 2005, and December 31, 2007, resulting in a diagnosis of ulcerative proctitis (ICD-9-CM 556.2) or ulcerative proctosigmoiditis (556.3)	11,475

Evidence of receipt of a 5-ASA (oral, enema, or suppository) within 30 days of this procedure (date of first receipt termed “index date”)	2587

≥12 months of complete encounter data before and after index date	1401

No evidence of receipt of a 5-ASA, metronidazole, antimetabolite, glucocorticoid, or TNF inhibitor in the 365 days preceding index date	743

No medical encounters with a primary diagnosis code for ulcerative colitis (ICD-9-CM 556.X, excluding 556.2 and 556.3) and/or Crohn's disease (ICD-9-CM 555.X or 556.X) 31 to 365 days prior to index date	693

No evidence of receipt of >1 UP-related medication on index date	548

UP: ulcerative proctitis.

**Table 2:** Baseline characteristics of study subjects with ulcerative proctitis (*N* = 548), by treatment group.

Characteristic	Treatment group			Total (*N* = 548)
	Oral 5-ASA (*n* = 138)	Mesalamine suppository (*n* = 327)	Mesalamine enema (*n* = 83)	
Age, years				
Mean (SD)	51.3 (16.1)	45.5 (15.3)	47.5 (14.9)	47.3 (15.6)
Median	51	45	45	47
Minimum	20	18	18	18
Maximum	89	89	86	89
Frequency distribution by age group, years, *n* (%)				
18–24	7 (5.1)	28 (8.6)	5 (6.0)	40 (7.3)
25–34	14 (10.1)	55 (16.8)	12 (14.5)	81 (14.8)
35–44	29 (21.0)	75 (22.9)	22 (26.5)	126 (23.0)
45–54	33 (23.9)	78 (23.9)	17 (20.5)	128 (23.4)
55–64	29 (21.0)	60 (18.3)	15 (18.1)	104 (19.0)
65–74	12 (8.7)	17 (5.2)	7 (8.4)	36 (6.6)
75–84	9 (6.5)	11 (3.4)	4 (4.8)	24 (4.4)
85–94	5 (3.6)	3 (0.9)	1 (1.2)	9 (1.6)
Gender, *n* (%)				
Male	77 (55.8)	136 (41.6)	39 (47.0)	252 (46.0)
Female	61 (44.2)	191 (58.4)	44 (53.0)	296 (54.0)
Geographic location, *n* (%)				
Northeast	17 (12.3)	42 (12.8)	13 (15.7)	72 (13.1)
North central	49 (35.5)	93 (28.4)	20 (24.1)	162 (29.6)
South	45 (32.6)	145 (44.3)	35 (42.2)	225 (41.1)
West	26 (18.8)	45 (13.8)	15 (18.1)	86 (15.7)
Unknown	1 (0.7)	2 (0.6)	0 (0.0)	3 (0.5)
Principal payer, *n* (%)				
Basic/major medical	0 (0.0)	0 (0.0)	0 (0.0)	0 (0.0)
Comprehensive	30 (21.7)	42 (12.8)	13 (15.7)	85 (15.5)
HMO	19 (13.8)	53 (16.2)	11 (13.3)	83 (15.1)
POS, POS with capitation	12 (8.7)	43 (13.1)	11 (13.3)	66 (12.0)
PPO	74 (53.6)	178 (54.4)	46 (55.4)	298 (54.4)
Other	3 (2.2)	11 (3.4)	2 (2.4)	16 (2.9)
Therapy days dispensed on index date, *n* (%)				
Oral 5-ASA	31 (15.2)	0 (0.0)	0 (0.0)	8 (15.6)
Mesalamine suppository	0 (0.0)	27 (9.0)	0 (0.0)	16 (14.8)
Mesalamine enema	0 (0.0)	0 (0.0)	22 (8.0)	3 (8.6)
Comorbidities (ICD-9-CM), *n* (%)				
Colorectal comorbidities (ICD-9-CM)				
Abscess of anal and rectal regions (566)	0 (0.0)	1 (0.3)	0 (0.0)	1 (0.2)
Anal fissure and fistula (565)	3 (2.2)	4 (1.2)	2 (2.4)	9 (1.6)
Benign tumor of the colon (211.3)	15 (10.9)	24 (7.3)	9 (10.8)	48 (8.8)
Benign tumor of the rectum and anal canal (211.4)	1 (0.7)	5 (1.5)	1 (1.2)	7 (1.3)
Diverticula of intestine (562)	10 (7.2)	12 (3.7)	6 (7.2)	28 (5.1)
Functional digestive disorders, not elsewhere classified (564)	15 (10.9)	36 (11.0)	6 (7.2)	57 (10.4)
Malignant neoplasm of colon (153)	0 (0.0)	0 (0.0)	1 (1.2)	1 (0.2)
Other and unspecified noninfectious gastroenteritis and colitis (558)	54 (39.1)	56 (17.1)	25 (30.1)	135 (24.6)
Vascular insufficiency of intestine (557)	2 (1.4)	0 (0.0)	0 (0.0)	2 (0.4)
Other digestive comorbidities (ICD-9-CM)				
Chronic liver disease and cirrhosis (571)	1 (0.7)	2 (0.6)	1 (1.2)	4 (0.7)
Diseases of esophagus (530)	13 (9.4)	30 (9.2)	9 (10.8)	52 (9.5)
Disease of pancreas (577)	0 (0.0)	2 (0.6)	0 (0.0)	2 (0.4)
Duodenal ulcer (532)	0 (0.0)	1 (0.3)	0 (0.0)	1 (0.2)
Gastric ulcer (531)	0 (0.0)	1 (0.3)	1 (1.2)	2 (0.4)
Common nondigestive comorbidities (ICD-9-CM)				
Asthma (493)	5 (3.6)	7 (2.1)	2 (2.4)	14 (2.6)
Atherosclerosis (440)	1 (0.7)	2 (0.6)	0 (0.0)	3 (0.5)
Chronic bronchitis (491)	2 (1.4)	0 (0.0)	0 (0.0)	2 (0.4)
Diabetes mellitus (250)	15 (10.9)	16 (4.9)	6 (7.2)	37 (6.8)
Essential hypertension (401)	29 (21.0)	52 (15.9)	17 (20.5)	98 (17.9)
Iron deficiency anemias (280)	10 (7.2)	5 (1.5)	2 (2.4)	17 (3.1)
Ischemic heart disease (410–414)	13 (9.4)	12 (3.7)	4 (4.8)	29 (5.3)

HMO: health maintenance organization.

Oral 5-ASA: oral 5-aminosalicylate.

POS: point of service.

PPO: preferred provider organization.

**Table 3:** Cumulative number of study subjects experiencing treatment escalation, by duration of follow-up and treatment group.

Treatment group	Month 3		Month 6		Month 9		Month 12	
	*n* (%)	95% CI (%)	*n* (%)	95% CI (%)	*n* (%)	95% CI (%)	*n* (%)	95% CI (%)
Oral 5-ASA (*n* = 138)	29 (21.0)	(14.2, 27.8)	37 (26.8)	(19.4, 34.2)	42 (30.4)	(0.3, 22.8)	47 (34.1)	(26.2, 42.0)
Mesalamine suppository (*n* = 327)	31 (9.5)	(6.3, 12.7)	52 (15.9)	(11.9, 19.9)	59 (18.0)	(0.2, 13.9)	68 (20.8)	(16.4, 25.2)
Mesalamine enema (*n* = 83)	10 (12.0)	(5.0, 19.1)	15 (18.1)	(9.8, 26.4)	17 (20.5)	(0.2, 11.8)	17 (20.5)	(11.8, 29.2)

Oral 5-ASA: oral 5-aminosalicylate.

**Table 4:** Cumulative incidence of hospitalization for inflammatory bowel disease, by treatment group and duration of follow-up.

Treatment group	Month 3 *n* (%)	Month 6 *n* (%)	Month 9 *n* (%)	Month 12 *n* (%)
Oral 5-ASA (*n* = 138)	3 (2.2)	4 (2.9)	4 (2.9)	5 (3.6)
Mesalamine suppository (*n* = 327)	4 (1.2)	5 (1.5)	5 (1.5)	6 (1.8)
Mesalamine enema (*n* = 83)	1 (1.2)	1 (1.2)	2 (2.4)	2 (2.4)

Oral 5-ASA: oral 5-aminosalicylate.

**Table 5:** Cumulative incidence of emergency department visits for inflammatory bowel disease, by treatment group and duration of follow-up.

Treatment group	Month 3 *n* (%)	Month 6 *n* (%)	Month 9 *n* (%)	Month 12 *n* (%)
Oral 5-ASA (*n* = 138)	1 (0.7)	1 (0.7)	1 (0.7)	1 (0.7)
Mesalamine suppository (*n* = 327)	3 (0.9)	4 (1.2)	4 (1.2)	6 (1.8)
Mesalamine enema (*n* = 83)	0 (0.0)	1 (1.2)	2 (2.4)	2 (2.4)

Oral 5-ASA: oral 5-aminosalicylate.

**Table 6:** Cumulative total per-patient cost (in USD) of pharmacotherapy related to inflammatory bowel disease, by treatment group and duration of follow-up.

Treatment group	Month 3 Mean $ (SD)	Month 6 Mean $ (SD)	Month 9 Mean $ (SD)	Month 12 Mean $ (SD)
Oral 5-ASA (*n* = 138)	589 (424)	926 (699)	1,251 (1,089)	1,552 (1,418)
Mesalamine suppository (*n* = 327)	460 (330)	663 (607)	815 (829)	986 (1,095)
Mesalamine enema (*n* = 83)	514 (369)	646 (596)	850 (929)	995 (1,172)

Oral 5-ASA: oral 5-aminosalicylate.

## Abbreviations

UP: Ulcerative proctitis
5-ASA: Oral 5-aminosalicylate
UP Rx: Mesalamine.

## References

[1] Bello C., Belaiche J., Louis E., Reenaers C. (2011). Evolution and predictive factors of relapse in ulcerative colitis patients treated with mesalazine after a first course of corticosteroids. *Journal of Crohn's and Colitis*.

[2] Meucci G., Vecchi M., Astegiano M. (2000). The natural history of ulcerative proctitis: a multicenter, retrospective study. *American Journal of Gastroenterology*.

[3] Kornbluth A., Sachar D. B., Practice Parameters Committee of the American College of Gastroenterology (2010). Ulcerative colitis practice guidelines in adults: American College of Gastroenterology, Practice Parameters Committee. *The American Journal of Gastroenterology*.

[4] Hanauer S. B., Sparrow M. (2004). Therapy of ulcerative colitis. *Current Opinion in Gastroenterology*.

[5] Regueiro M., Loftus E. V., Steinhart A. H., Cohen R. D. (2006). Clinical guidelines for the medical management of left-sided ulcerative colitis and ulcerative proctitis: summary statement. *Inflammatory Bowel Diseases*.

[6] Karagozian R., Burakoff R. (2007). The role of mesalamine in the treatment of ulcerative colitis. *Therapeutics and Clinical Risk Management*.

[7] Gionchetti P., Rizzello F., Venturi A. (1998). Comparison of oral with rectal mesalazine in the treatment of ulcerative proctitis. *Diseases of the Colon and Rectum*.

[8] Kam L., Cohen H., Dooley C., Rubin P., Orchard J. (1996). A comparison of mesalamine suspension enema and oral sulfasalazine for treatment of active distal ulcerative colitis in adults. *American Journal of Gastroenterology*.

[9] Biddle W. L., Greenberger N. J., Swan J. T., McPhee M. S., Miner P. B. (1988). 5-Aminosalicylic acid enemas: effective agent in maintaining remission in left-sided ulcerative colitis. *Gastroenterology*.

[12] Cohen R. D., Woseth D. M., Thisted R. A., Hanauer S. B. (2000). A meta-analysis and overview of the literature on treatment options for left-sided ulcerative colitis and ulcerative proctitis. *American Journal of Gastroenterology*.

[13] Harris M. S., Lichtenstein G. R. (2011). Review article: delivery and efficacy of topical 5-aminosalicylic acid (mesalazine) therapy in the treatment of ulcerative colitis. *Alimentary Pharmacology and Therapeutics*.

[14] Bressler B., Marshall J. K., Bernstein C. N. (2015). Clinical practice guidelines for the medical management of nonhospitalized ulcerative colitis: the Toronto consensus. *Gastroenterology*.

[15] Safdi M., DeMicco M., Sninsky C. (1997). A double-blind comparison of oral versus rectal mesalamine versus combination therapy in the treatment of distal ulcerative colitis. *American Journal of Gastroenterology*.

[16] Thirumurthi S., Chowdhury R., Richardson P., Abraham N. S. (2010). Validation of ICD-9-CM diagnostic codes for inflammatory bowel disease among veterans. *Digestive Diseases and Sciences*.

